# COVID-19: Contrasting experiences of South African physiotherapists based on patient exposure

**DOI:** 10.4102/sajp.v78i1.1576

**Published:** 2022-01-11

**Authors:** Tasneem Hassem, Nicky Israel, Nabeelah Bemath, Tarique Variava

**Affiliations:** 1 Department of Psychology, Faculty of Humanities, University of the Witwatersrand, Johannesburg, South Africa; 2 School of Anatomical Sciences, Faculty of Health Sciences, University of the Witwatersrand, Johannesburg, South Africa

**Keywords:** mental health, COVID-19 pandemic, physiotherapists, South Africa, well-being

## Abstract

**Background:**

The coronavirus disease 2019 (COVID-19) pandemic has exposed physiotherapists to unique work-related challenges. However, there is a lack of research regarding the mental health and lived experiences of South African physiotherapists during the COVID-19 pandemic.

**Objectives:**

To determine levels of mental and physical health, burnout, depression, anxiety and resilience and coping strategies used by a sample of South African physiotherapists with and without exposure to patients with COVID-19. Lived work experience, perceived health and sources of support were also explored.

**Method:**

A non-experimental, cross-sectional, mixed-method design was used. Physiotherapists completed an online survey comprising: a demographic questionnaire; scales assessing mental and physical health, burnout, depression, anxiety and coping strategies and six open-ended questions. A total of 171 physiotherapists participated in our study, 43.3% of whom were exposed to patients with COVID-19.

**Results:**

The exposure group scored significantly higher on self-reported mental health, anxiety, depression and burnout than the non-exposure group. No significant differences were reported for physical health and resilience. Significantly more maladaptive coping strategies were employed by the exposure group. Participants’ lived experiences highlighted similar experiences, as well as work-related challenges. Both groups reported that primary sources of support were significant others, but highlighted the lack of organisational support.

**Conclusion:**

Irrespective of the degree of exposure to COVID-19, the mental health and lived experiences of physiotherapists working in South Africa has been negatively impacted by COVID-19.

**Clinical implications:**

Understanding physiotherapists’ well-being and lived experiences during the pandemic may guide workplace interventions. Our findings suggest the need for psycho-educational intervention programmes to be implemented at an organisational level.

## Introduction

On 11 March 2020, the World Health Organization (WHO) (2020a) declared the outbreak of coronavirus disease 2019 (COVID-19) as a pandemic. This resulted in many countries undergoing nationwide lockdowns – including South Africa (South African Government 2020a). Notably, healthcare workers (HCWs) were exempted from lockdown regulations given their essential role in the fight against COVID-19. The WHO (2020b) has highlighted that the increased workload, situational anxiety, witnessing of death and risk of infection faced by HCWs during the pandemic could influence their mental health and quality of life.

Physiotherapists play a unique role in the management and treatment of COVID-19 patients. Both globally, as well as in South Africa, physiotherapists have been actively involved in addressing COVID-19-related cardio-respiratory conditions, pain and musculoskeletal dysfunction, neurological conditions and patient mental health and well-being (South African Society of Physiotherapy [SASP] 2020; Thomas et al. 2020). Physiotherapists like other HCWs are therefore at an increased risk of mental health challenges. However, research on the stressors experienced by physiotherapists during COVID-19 is lacking in a South African context.

Research conducted on the mental health of HCWs during the first global peak of the COVID-19 pandemic highlighted that frontline HCWs experienced increased symptoms of depression, anxiety and psychological stress (Vizheh et al. 2020; Zhang et al. 2020). However, HCWs who worked with COVID-19 patients were identified as having an elevated risk of developing mental health complications when compared with HCWs who did not (Badahdah et al. 2020; Di Tella et al. 2020; Lu et al. 2020). A systematic review on the mental health of HCWs during the COVID-19 pandemic reported that the sample of HCWs used consisted primarily of nurses and physicians (Vizheh et al. 2020), highlighting the lack of research carried out on physiotherapists.

In addition to the risks physiotherapists may face as frontline HCWs, COVID-19 has presented stressors related to patient care and adjustment to amended roles and activities as a result of the pandemic, including the wearing of personal protective equipment (PPE) (Aderonmu 2020; Falvey, Krafft & Kornetti 2020; Righetti et al. 2020; Thomas et al. 2020). Coronavirus disease 2019 has also caused disruption in physiotherapists’ work activity because of challenges in practising contact therapy whilst minimising the risk of infection, subsequently negatively impacting the financial circumstances of physiotherapists and their families (Aderonmu 2020; Minghelli et al. 2020).

Physiotherapists within the South African context have faced similar challenges. At the start of the outbreak, the SASP (2020) advised that all outpatient physiotherapy departments and practices reschedule appointments with low-risk patients and manage severe and acute risk patients with little-to-no contact. The financial impact of COVID-19 on physiotherapists was echoed in a letter published by the SASP during the initial lockdown in South Africa (SASP 2020). Physiotherapists working in the South African public health system face additional unique stressors as they are located within a context of a fragmented, under-resourced and overburdened healthcare system (Coovadia et al. 2009).

Given these unique factors such as financial burden and changing roles of physiotherapists during the pandemic, coupled with being amongst the frontline workers treating COVID-19 patients, there is an urgent need to assess the well-being and lived experiences of physiotherapists practising in South Africa.

Our study aimed to determine levels of physical and mental health, depression, anxiety, burnout and resilience, as well as coping strategies used in a sample of physiotherapists working in South Africa during the COVID-19 pandemic. Differences between those with and without COVID-19 exposure (treatment of or regular contact with COVID-19 patients) were also explored as were lived work experiences, perceived health and sources of support.

## Method

Our study adopted a cross-sectional, non-experimental and mixed-method design (Creswell & Plano Clark 2017; Stangor 2015), where an online survey was used to obtain quantitative and qualitative data regarding the mental health and experiences of physiotherapists in South Africa during the COVID-19 pandemic. Qualitative and quantitative data were analysed separately; but were integrated for interpretation.

### Setting

Data were collected from physiotherapists across South Africa in June and July 2020. This coincided with the first Alert Level 3 lockdown implemented by the South African government during the pandemic. This alert level signified that there was a moderate rate of transmission of COVID-19 and moderate capacity and readiness of the health system. It allowed for the opening of various socioeconomic sectors subject to the stricter restrictions imposed during alert Levels 4 and 5 lockdown. These included curfews, constraints on travel and public and social gatherings and prescriptive safety measures such as the wearing of masks and social distancing (South African Government 2020a, 2020b).

### Study population and sampling strategy

The target population for our study was qualified physiotherapists practising in South Africa during the pandemic, that is, individuals who had completed at least an undergraduate professional degree in Physiotherapy (including physiotherapists serving their community service). Volunteer participants were recruited using a non-probability, convenience sampling strategy (Laher & Botha 2012).

The link to the online survey was sent to the SASP with a request to disseminate this to all members. The link was also circulated on various social media platforms and to some hospitals. Individuals who received the invitation, consented to participate and completed the survey, constituted the final sample of 171 qualified physiotherapists.

### Data collection

The online survey consisted of a demographic questionnaire, a brief physical health questionnaire, mental health screening instruments and six open-ended questions.

#### Demographic questionnaire

The demographic questionnaire requested participants’ gender, age, level of education, work experience, relationship structures, health conditions and exposure to COVID-19.

#### Global Health Indicators

A single item drawn from the Global Physical Health Scale (GPH-4) and a single item drawn from the Global Mental Health Scale (GMH-4) (Hays et al. 2017) were used to capture participants’ self-rated levels of physical and mental health, respectively. Each item was answered on a five-point Likert-type scale ranging from ‘poor’ to ‘excellent’. Formal reliability cannot be established for single item measures. However, internal consistency reliability estimates for different versions of the GPH-4 and the GMH-4 scales have been shown to range from moderate to high, and briefer versions have been shown to be psychometrically sound (Hays et al. 2017).

#### Hospital Anxiety and Depression Scale

The Hospital Anxiety and Depression Scale (HADS) was used to measure anxiety and depression. It contains two subscales, each of which contains seven items rated on a four-point scale with different anchor points (Snaith 2003). The HADS has been found to be valid across different languages and contexts, including in community settings (Bjelland et al. 2002; Snaith 2003). In our study, Cronbach’s alpha for the depression subscale was 0.809 and for the anxiety subscale was 0.876.

#### Burnout Measure, Short Version

The Burnout Measure Short Version (BMS) was used to measure symptoms of emotional, physical and mental exhaustion representing burnout (Malach-Pines 2005). It contains 10 items rated on a seven-point scale (‘never’ to ‘always’). The measure has been found to be reliable and valid (Fatoki 2019; Malach-Pines 2005). The BMS was found to have a Cronbach’s alpha of 0.912 in our study.

#### The Connor-Davidson Resilience Scale

The Connor-Davidson Resilience Scale (CD-RISC) assesses resilience or the extent of a respondent’s ability to adapt well during significant stressful circumstances (Connor & Davidson 2003). Our study used the CD-RISC-10 (Vaishnavi, Connor & Davidson 2007), consisting of 10 items answered on a four-point scale (‘not true at all’ to ‘true nearly all of the time’). The CD-RISC-10 has been shown to be a valid measure of resilience (Vaishnavi et al. 2007) and had a Cronbach’s alpha of 0.879 in our study.

#### The Brief COPE Inventory

Coping strategies were assessed using the Brief COPE Inventory (Carver 1997). This scale contains 28 items divided into 14 subscales, each of which is answered on a four-point scale (‘I haven’t been doing this’ to ‘I’ve been doing this a lot’). The inventory has been found to demonstrate acceptable reliability and a factor structure that closely matches to that of the full inventory (Carver 1997). In our study, Cronbach’s alphas for five of the subscales (self-distraction, active coping, denial, venting and acceptance) fell below 0.60; these subscales were thus excluded from subsequent analysis. Cronbach’s alphas for the remaining nine subscales ranged from 0.679 (behavioural disengagement) to 0.951 (substance use), indicating acceptable internal consistency reliability (Hair et al. 2006). Scores from all of the subscales were also combined to form two broader coping indices – adaptive coping (positive or constructive coping strategies) and maladaptive coping (negative or destructive coping strategies). The Cronbach’s alphas for these indices in our study were 0.825 and 0.742, respectively.

#### Open-ended questions

At the end of the survey, participants were asked six open-ended questions about their experiences since COVID-19 began. These questions explored topics such as their experiences at work and any challenges they experienced, their perceived physical and mental health, their support structures at work and at home and their comments to the South African Minister of Health.

### Data analysis

The SPSS (Statistical Package for the Social Sciences) software (IBM Corp. 2020) was used to analyse the quantitative data following entry into Microsoft Excel and cleaning. Internal consistency reliability estimates for the instruments were calculated, as were descriptive statistics for all of the main variables in the overall sample (Field 2013). In cases where parametric assumptions were met, independent sample *t*-tests were carried out to establish whether there were significant differences in levels of the main variables between the group of physiotherapists in the sample with COVID-19 exposure and those without exposure. In instances where parametric assumptions were violated (specifically normality), non-parametric Mann–Whitney U tests were used (Field 2013). Effect sizes were calculated using Cohen’s *d* or *r* estimates as appropriate (Field 2013). Qualitative data obtained from the open-ended questions was analysed using traditional content analysis (Hsieh & Shannon 2005). Inductive categories were developed through a process of preliminary coding, followed by category development, clustering and relative weighting based on frequency (Hsieh & Shannon 2005). Comparisons of the frequencies between the groups with and without COVID-19 exposure were built into the content analysis. Peer debriefing occurred throughout the analysis to ensure trustworthiness.

### Ethical considerations

Ethical approval was obtained from the University of the Witwatersrand Ethics Committee, clearance certificate number: M200461. Participants were informed of our study’s particulars through an information sheet and remained anonymous unless they provided their details to be contacted for a follow-up interview.

## Results

The final sample consisted of 171 registered physiotherapists, 43.3% of whom had either treated or had regular contact with COVID-19 patients (the COVID-19 exposure group). The average age of the sample was 37.25 years (standard deviation [SD] = 11.28; range: 22–70). The majority of the sample was female (95.3%); English-speaking (60.2%) and had an undergraduate degree (82.4%; see Table 1). Most participants were in a relationship (71.3%) and lived with either a partner or a partner and children (68.4%). The majority of the sample did not have a chronic condition (78.3%) and did not use chronic medication (64.9%). On average, the sample participants had 14.43 years of experience working as a physiotherapist (SD = 11.26; range: 1–42). Relative to sample size, the demographic profiles of the exposure and non-exposure groups were fairly similar (see Table 1).

**TABLE 1:** Demographic characteristics of the sample: Overall and by group.

Variable	Category	Overall Sample		No COVID-19 exposure		COVID-19 exposure	
		*N*	%	*N*	%	*N*	%
Total	-	171	100.0	97	56.7	74	43.3
Gender (*n* = 170)	Female	163	95.3	92	53.8	71	41.5
	Male	7	4.1	5	2.9	2	1.2
Home language(*n* = 168)	Afrikaans	58	33.0	29	17.0	29	17.0
	English	103	60.2	64	37.4	39	22.8
	isiXhosa	3	1.8	1	0.6	2	1.2
	Sepedi	4	2.3	3	1.8	1	0.6
Level of education (*n* = 170)	Undergraduate degree	141	82.4	79	46.2	62	36.2
	Master’s degree	29	17.0	17	9.9	12	7.1
Religious affiliation	Christianity	134	78.3	81	47.4	53	30.9
	Hinduism	6	3.5	1	0.6	5	2.9
	Islam	8	4.7	2	1.2	6	3.5
	Judaism	2	1.2	1	0.6	1	0.6
	No religion	17	9.9	11	6.4	6	3.5
	Other	4	2.3	1	0.6	3	1.8
Relationship status (*n* = 170)	No	48	28.1	25	14.6	23	13.5
	Yes	122	71.3	71	41.5	51	29.8
Marital status	No	76	44.4	33	19.3	43	25.1
	Yes	95	55.6	64	37.4	31	18.2
Number of children (*n* = 169)	0	86	50.3	40	23.4	46	26.9
	1	25	14.6	18	10.5	7	4.1
	2	45	26.3	30	17.5	15	8.8
	3	10	5.9	7	4.1	3	1.8
	4	3	1.8	1	0.6	2	1.2
Living condition	Alone	20	11.7	9	5.3	11	6.4
	With a partner	55	32.2	32	18.7	23	13.5
	With a partner and children	62	36.2	41	23.9	21	12.3
	With children	4	2.3	3	1.8	1	0.6
	With immediate family	25	14.6	11	6.4	14	8.2
	With other relatives	5	2.9	1	0.6	4	2.3
Chronic condition	No	134	78.3	74	43.3	60	35.0
	Yes	37	21.7	23	13.5	14	8.2
Chronic medication (*n* = 170)	No	111	64.9	63	36.8	48	28.1
	Yes	59	34.5	33	19.3	26	15.2

### Levels of mental and physical health, burnout, depression, anxiety, resilience and coping strategies in COVID-19 exposure and non-exposure groups

It can be observed from Table 2 that the average self-reported physical health was high in the sample with no significant difference between the exposure groups. In contrast, there was a significant difference in levels of self-reported mental health between the groups (*t* = 3.292; *p* = 0.001; *d* = 0.501), where participants with exposure reported lower levels of mental health. Significant differences were also observed for levels of anxiety (*t* = –3.405; *p* = 0.001; *d* = 0.524), depression (*t* = –2.769; *p* = 0.006; *d* = 0.427) and burnout (*t* = –3.733; *p* = 0.000; *d* = 0.581); these were higher for participants with exposure. There were no significant differences in reported resilience or adaptive coping strategy use between the groups. However participants with exposure reported using significantly more maladaptive coping strategies (*z* = –2.125; *p* = 0.034; *r* = 0.163), specifically self-blame (*z* = –2.401; *p* = 0.016; *r* = 0.184) and behavioural disengagement (*z* = –2.468; *p* = 0.014; *r* = 0.189).

**TABLE 2:** Descriptive statistics and comparisons between the groups.

Variable	Overall sample (*n* = 171)				No COVID-19 exposure (*n* = 97)		COVID-19 exposure (*n* = 74)		Test Stat.	Effect size
	M	SD	Min.	Max.	M	SD	M	SD		
Physical health	3.89	0.805	2	5	3.95	0.821	3.82	0.783	0.999	0.162
Mental health	3.50	1.002	1	5	3.71	0.946	3.22	1.010	3.292***	0.501
Anxiety	8.89	4.335	0	21	7.94	4.135	10.15	4.296	−3.405***	0.524
Depression	5.77	3.573	0	17	5.12	3.474	6.62	3.545	−2.769**	0.427
Resilience	28.36†	6.122	11	40	29.14§	5.581	27.34¶	6.671	1.900	0.293
Burnout	32.13‡	11.651	10	67	29.34	11.477	35.84¶	10.888	−3.733***	0.581
Adaptive coping	30.19	9.096	1	52	29.87	9.918	30.61	7.936	0.599	0.082
Maladaptive coping	4.02	3.676	0	17	3.46	3.311	4.74	4.014	−2.125*	0.163
Emotional support	2.74†	1.691	0	6	2.70§	1.784	2.79¶	1.572	0.714	0.054
Instrumental support	2.56	1.652	0	6	2.48	1.690	2.66	1.607	0.488	0.109
Positive reframing	3.13	1.673	0	6	3.08	1.663	3.20	1.696	0.643	0.071
Planning	3.51	1.660	0	6	3.54	1.738	3.49	1.564	0.847	0.030
Humour	1.89‡	1.584	0	6	1.83§	1.540	1.97	1.647	0.570	0.088
Religion	3.38	2.140	0	6	3.55	2.189	3.16	2.068	0.246	0.183
Substance use	0.60	1.176	0	6	0.57	1.136	0.64	1.234	−0.252	0.019
Self-blame	1.60	1.675	0	6	1.32	1.504	1.96	1.824	−2.401*	0.184
Behavioural disengagement	0.98‡	1.317	0	6	0.78§	1.224	1.24	1.393	−2.468*	0.189

* , *p* < 0.05;

** , *p* < 0.01;

*** , *p* < 0.001.

† , *n* = 169;

‡ , *n* = 170;

§ , *n* = 96;

¶ , *n* = 73.

### Lived work experiences, perceived health and sources of support

Findings from the qualitative data were categorised into two main themes: (1) experiences and challenges at work, and (2) self-reported health and sources of support.

### Experiences and challenges at work

For both participants with no COVID-19 (NC) exposure (*n* = 91) and those with exposure (C) (*n* = 67), one of the most frequent work changes reported (by 58.2% of the non-exposure group and 31.3% of the exposure group) was a large reduction in patient load accompanied by a loss of income and increased operating costs:

‘Work has dropped dramatically. Low client loads and thus financial stress…’ (NC34, Female, 33 years old)

‘…increasing costs of required PPE and taxation are big worries…’ (NC91, Female, 45 years old)

‘Work is very quiet…I have a big decrease in income…’ (C2, Female, 24 years old)

Additional time, stress and workload demands associated with wearing PPE and new hygiene protocols (reported by 27.5% of the non-exposure group and 20.9% of the exposure group) was another commonly reported change:

‘…much more demanding because of COVID-19 protocols…’ (NC69, Male, 64 years old)

‘…more time needs to be incorporated to sanitise between patients…’ (C28, Female, 38 years old).

Both groups also observed increased stress, burnout, emotional exhaustion, anxiety and fear related to transmission of COVID-19 (reported by 27.5% of the non-exposure group and 31.3% of the exposure group) and more emotional pressure in the work environment as a result of needing to manage fear and panic experienced by patients and colleagues and offer them support (reported by 24.2% of the non-exposure group and 20.9% of the exposure group) as common changes:

‘…a bit scary in case I contract corona or pass it on… dealing with a lot of patients and co-worker anxiety.’ (NC32, Female, 56 years old)

‘Tense; general panic …’ (C63, Female, 32 years old)

‘…treating patients has become more and more emotionally draining…’ (NC86, Female, 32 years old)

Participants with COVID-19 exposure raised unique work changes related to ineffective management, changing work roles and a lack of clarity regarding regulations (reported by 26.9% of the group) and an increase in workload and pressure to perform under conditions with high demand and insufficient staffing (raised by 25.4% of the group):

‘It has been stressful because of staff shortages…and stress from management making decisions that are not in the best interest of staff and patients and overworking staff… The rules/guidelines also change very regularly, making it hard to keep up…’ (C41, Female, 26 years old)

‘Slightly abnormal with a lot more regulations and changes…at times the rules made no sense and people felt frustrated.’ (C33, Female, 29 years old)

‘Having to perform at such a high intensity for long periods.’ (C38, Female, 36 years old)

These changes were only raised by a single participant in the non-exposure group (1.1%). Similarly, a loss of everyday work routine was observed as a meaningful change by the non-exposure group (reported by 20.9% of the group) but was not raised by many participants in the exposure group (reported by only 6%). A small percentage of the exposure group (11.9%) also reported feeling increased pride and satisfaction in their work, which was not raised by any of the participants in the non-exposure group. These differences could possibly reflect distinctions in the types of work environments between the groups (although not formally assessed in our study, more participants in the exposure group mentioned working in a hospital environment and more participants in the non-exposure group mentioned being in private practice).

The most notable work challenges raised by the non-exposure group were loss of work (reported by 41.8%), loss of income and reduced financial stability (reported by 35.2%), implementation of safety protocols (reported by 22.0%), time management linked to administrative load and new procedures (reported by 17.6%), providing appropriate patient support (reported by 12.1%) and managing risk of exposure to COVID-19 (reported by 12.1%):

‘Praying that we see enough patients to pay our bills and salaries… patients feeling hopeless…’ (NC2, Female, 40 years old)

‘…to make sure that we maintain very stringent sanitising protocols…’ (NC30, Female, 64 years old)

‘…administrative burden on business efficiency which will affect longevity and sustainability of the practice…’ (NC6, Female, 32 years old)

‘The stress of either getting COVID-19 or passing it on…’ (NC14, Female, 33 years old)

The most notable work challenges raised by the exposure group were: time management linked to increased work intensity and new responsibilities (reported by 25.4%), implementation of safety protocols (reported by 22.4%), access to PPE (reported by 20.9%), lack of leadership and managerial support (reported by 19.4%), managing risk of exposure to COVID-19 (reported by 19.4%), reduced patient load (reported by 17.9%) and financial challenges (reported by 11.9%):

‘Lack of resources. Absence of leadership from … management. Staff is fearful of contracting COVID-19 but behave irresponsibly…’ (C23, Female, 52 years old)

‘Increased workload, policies changing almost daily…’ (C37, Female, 27 years old)

‘Loss of income. Regular possible exposure.’ (C19, Female, 49 years old)

The exposure group also raised insufficient information-sharing and communication from management and between colleagues and feelings of hopelessness, burnout, guilt, fear and trauma as significant challenges (reported by 16.4%), These challenges were raised by a much smaller percentage of non-exposure participants also (reported by 5.5%). Insufficient staffing as a result of the need to quarantine was also raised as an almost unique challenge in the exposure group (reported by 13.4% compared with only 1.1% in the non-exposure group). Other challenges raised included: providing support for patients (reported by 12.1% in the non-exposure group and 6.0% in the exposure group), stressful or reduced peer interactions (reported by 4.4% in the non-exposure group and 11.9% in the exposure group), non-adherence to protocols (reported by 4.4% in the non-exposure group and 9.0% in the exposure group) and discomfort and communication barriers associated with wearing PPE (reported by 6.6% in the non-exposure group and 4.5% in the exposure group).

### Self-reported health and sources of support

Over half of the participants in the non-exposure group (65.9%) reported that their health was good or excellent; on the contrary, this was reported by less than half of the participants in the exposure group (41.8%). In addition, only a very small percentage of the non-exposure group (3.3%) reported that their health was poor or had been impacted negatively compared to a larger percentage of the exposure group (14.9%). In the exposure group, participants reported experiencing fatigue and exhaustion (25.4%), impaired mental health, anxiety or burnout (13.4%), feeling unwell (10.4%), headaches or migraine (9.0%) and mild illness (9.0%). These same symptoms were reported far less by those in the non-exposure group (9.9% for fatigue and exhaustion, 4.4% for impaired mental health, anxiety or burnout, 0% for feeling unwell, 4.4% for headaches or migraine and 4.4% for mild illness).

Across both groups, the primary sources of personal support reported were spouses and family (84.6% for the non-exposure group and 89.6% for the exposure group), friends and room-mates (17.6% for the non-exposure group and 20.9% for the exposure group), religion (7.7% for the non-exposure group and 4.5% for the exposure group), and sports, exercise and hobbies (6.6% for the non-exposure group and 3.0% for the exposure group). In the work environment, the primary sources of support reported were: work colleagues and teams (49.5% for the non-exposure group and 65.7% for the exposure group); workplace interventions, counselling and training (13.2% for the non-exposure group and 25.4% for the exposure group) and management or the organisation (8.8% for the non-exposure group and 11.9% for the exposure group). It was of concern, however, that a relatively large percentage of physiotherapists in both groups (41.8% for the non-exposure group and 25.4% for the exposure group) indicated that they received no or very minimal support in their work environment. The SASP was mentioned as a source of support in the work environment by a small percentage of participants in both groups (3.3% in the non-exposure group and 3.0% in the exposure group).

Participants in both groups were also asked whether there was any feedback they would provide to the Minister of Health if they were afforded this opportunity. The exposure group provided a more cohesive set of feedback for this question. The most commonly raised issues included: greater provision of PPE and assistance with associated costs (23.9%), provision of more support for HCWs (17.9%), recognition of the unreasonable nature of current expectations and the negative effects for staff (16.4%), increased communication and information-sharing and improved guidelines for practice (13.4%) and improved disaster management planning and public healthcare provision (13.4%):

‘We need resources!’ (C57, Female, 25 years old)

‘Invest in healthcare workers by appointing staff and procuring PPE.’ (C23, Female, 52 years old)

‘Please supply us as healthcare workers with more information.’ (C49, Female, 28 years old)

‘The disaster plan put in place was not enough planning for this pandemic…’ (C1, Female, 29 years old)

The non-exposure group raised a more varied set of issues for this question. The most common issues they raised included: better public messaging and improved education around the pandemic (15.4%), stopping lockdown measures (9.9%), increased communication and information-sharing and improved guidelines for practice (9.9%), greater provision of PPE and assistance with associated costs (8.8%), addressing staff shortages (8.8%) and improved disaster management planning and public healthcare provision (8.8%):

‘Please continue to spread the message of how serious this is and ensure people follow protocols.’ (NC27, Female, 39 years old)

‘Be aware that most hospitals cannot cope and staff are inadequately prepared…’ (NC89, Female, 62 years old)

These sets of issues, particularly when considered in conjunction with patterns identified in responses for the other questions, served to highlight some of the distinctions in work experiences reported by physiotherapists in the exposure and non-exposure groups.

## Discussion

The findings of our study revealed that whilst there were no differences in self-reported levels of physical health or resilience between the groups, South African physiotherapists with COVID-19 exposure in the sample reported significantly reduced levels of mental health and increased levels of anxiety, depression and burnout when compared with those without COVID-19 exposure. Participants in the exposure group also reported experiencing more physical symptoms associated with physical and/or mental illness, especially fatigue. These findings were echoed in the qualitative accounts provided by the exposure group and align strongly with findings from international literature (Badahdah et al. 2020; Di Tella et al. 2020; Lu et al. 2020; Vizheh et al. 2020). Furthermore, whilst there were no differences reported between the groups for use of adaptive coping strategies, the exposure group reported an increased use of maladaptive coping strategies, specifically self-blame and behavioural disengagement. This has important implications for potential intervention, as the use of maladaptive coping strategies has been linked to reduced levels of mental health and thus finding effective ways to shift coping mechanisms to more adaptive forms may create a buffer against the negative mental health effects of COVID-19 patient exposure for physiotherapists (Moritz et al. 2016; Vizheh et al. 2020; Zhu et al. 2020).

Although concerns in context to loss of income were expressed slightly more strongly by the non-exposure group, both groups of physiotherapists in the sample highlighted reduced patient load and loss of income; additional stress associated with using PPE and implementing hygiene protocols; and increased emotional distress for self and others as primary work experiences during the pandemic. Work challenges identified for groups also included loss of work and income, implementation of new hygiene and protective protocols and the risk of contracting COVID-19 and associated anxiety. For the exposure group, however, there were a number of additional challenges linked directly to the treatment of COVID-19 patients, specifically access to PPE, a lack of clear guidance and support from leadership, insufficient information-sharing, insufficient staffing, increased workload and poor mental health. All of these concerns and experiences are directly in line with working conditions reported across the world for physiotherapists and HCWs generally during COVID-19 and serve to clearly illuminate both the potential detrimental personal effects of working with COVID-19 patients for HCWs in South Africa, including physiotherapists and the more general negative consequences of the pandemic for working professionals, including the field of physiotherapy in South Africa (Badahdah et al. 2020; Di Tella et al. 2020; Falvey et al. 2020; Lu et al. 2020; Minghelli et al. 2020; SASP 2020b; Vizheh et al. 2020).

Although physiotherapists in the sample reported multiple sources of personal support, especially family and friends, it was of considerable concern that across both groups, a significant number of participants reported experiencing a lack of support or limited support in their work environment, particularly from management. This lack of organisational support is concerning as research has shown that this can lower COVID-19-related anxiety (Zhu et al. 2020). This finding thus further enforces the urgent need for interventions to mitigate the negative effects of COVID-19 for physiotherapists in South Africa. This need was further illustrated in the suggestions put forward by participants for the Minister of Health, particularly those from the exposure group. These included: increased communication and better guidelines and information-sharing, improved disaster management protocols, greater provision of PPE and increased staffing.

Taken together, the findings regarding the reported mental health effects of the pandemic, challenges that have emerged as a result of this and the current structures in place in the work environment strongly support the development and implementation of both educational and mental health interventions for physiotherapists in South Africa. This is directly in line with international findings from similar studies (Badahdah et al. 2020; Di Tella et al. 2020; Lu et al. 2020; Vizheh et al. 2020; Zhu et al. 2020), and thus contributes to an emerging picture for best international practice in mitigating the effects of the pandemic for physiotherapists and HCWs more generally. Furthermore, the findings support similar forms of intervention to those recommended internationally, including: online interventions, education, and/or training designed to facilitate accurate information distribution; more effective communication; improved mental health; acute trauma exposure management; peer support from immediate colleagues and the broader profession; access to professional psychological services and the implementation and consistent enforcement of safety protocols designed to reduce risks because of COVID-19 exposure (Lu et al. 2020; Vizheh et al. 2020; Zhu et al. 2020). The findings also specifically supported the implementation of a mental health intervention designed to reduce maladaptive coping strategy use, particularly self-blame and behavioural disengagement, in South African physiotherapists with exposure to COVID-19 patients.

Whilst our study offers insight into the profile of physiotherapists working in South Africa during the COVID-19 pandemic, various limitations must be acknowledged. Firstly, the design of our study was non-experimental; this precludes drawing any causal conclusions regarding the links between COVID-19 patient exposure and mental health. Analysis of the potentially complex roles played by variables such as years of experience, relationship status and living condition was also beyond the scope of our study. Further research is thus required to determine predictive and causal factors linked to reduced mental health in South African physiotherapists treating COVID-19 patients and to refine the nature of potential interventions. In addition, the sample obtained was relatively small and did not fully represent the larger population of physiotherapists in South Africa. Additional research with larger and more representative samples is thus warranted. Findings from our study also suggested that there could be critical differences in the experiences of physiotherapists based on the nature of their place of work (e.g. in a hospital environment or in private practice) and based on the type of patient being treated and intensity of the treatment required. However, this information was not captured during data collection for our study. This is important information that should be gathered for future research, and that, merits further exploration in terms of refining understandings of physiotherapists’ experiences both within the current context of COVID-19 and post-pandemic. Lastly, the physiotherapist profile reported in our study only highlights experiences during the first Alert Level 3 lockdown in South Africa; further research is needed to explore changes in experiences and mental health as a result of the pandemic over time.

## Conclusion

The findings of our study suggest that the well-being of physiotherapists in South Africa has changed as a result of the COVID-19 pandemic, particularly for those with exposure to COVID-19 patients. This strongly supports the need for employers and managers to implement various psycho-educational interventions and programmes to foster improved work experiences and mental health amongst all South African physiotherapists (and physiotherapists and HCWs more generally), irrespective of level of exposure to COVID-19.
